# Participation and performance trends in ultra-endurance running races under extreme conditions - ‘Spartathlon’ versus ‘Badwater’

**DOI:** 10.1186/2046-7648-2-15

**Published:** 2013-05-01

**Authors:** Kristina da Fonseca-Engelhardt, Beat Knechtle, Christoph Alexander Rüst, Patrizia Knechtle, Romuald Lepers, Thomas Rosemann

**Affiliations:** 1 Institute of General Practice and for Health Services Research, University of Zurich, Zurich, 8091, Switzerland; 2 Gesundheitszentrum St. Gallen, St. Gallen, 9000, Switzerland; 3 INSERM U1093, Faculty of Sport Sciences, University of Burgundy, Dijon, Cedex, 21078, France; 4 Facharzt FMH für Allgemeinmedizin, Gesundheitszentrum St. Gallen, Vadianstrasse 26, St. Gallen, 9001, Switzerland

**Keywords:** Running, Ultra-endurance, Extreme conditions, Age, Sex differences

## Abstract

**Background:**

The aim of the present study was to compare the trends in participation, performance and age of finishers in ‘Badwater’ and ‘Spartathlon’ as two of the toughest ultramarathons in the world of more than 200 km of distance.

**Methods:**

Running speed and age of male and female finishers in Badwater and Spartathlon were analyzed from 2000 to 2012. Age of peak performance and sex difference in running speed were investigated during the studied period.

**Results:**

The number of female and male finishes increased in Badwater and Spartathlon. Women accounted on average for 21.5% ± 6.9% in Badwater and 10.8% ± 2.3% in Spartathlon. There was a significant increase in female participation in Badwater from 18.4% to 19.1% (*p* < 0.01) and in Spartathlon from 11.9% to 12.5% (*p* = 0.02). In men, the age of finishers was higher in Badwater (46.5 ± 9.3 years) compared to Spartathlon (44.8 ± 8.2 years) (*p* < 0.01). The age of female finishers of both races was similar with 43.0 ± 7.5 years in Badwater and 44.5 ± 7.8 years in Spartathlon (*p* > 0.05). Over the years, the age of the annual five fastest men decreased in Badwater from 42.4 ± 4.2 to 39.8 ± 5.7 years (*p* < 0.05). For women, the age remained unchanged at 42.3 ± 3.8 years in Badwater (*p* > 0.05). In Spartathlon, the age was unchanged at 39.7 ± 2.4 years for men and 44.6 ± 3.2 years for women (*p* > 0.05). In Badwater, women and men became faster over the years. The running speed increased from 7.9 ± 0.7 to 8.7 ± 0.6 km/h (*p* < 0.01) in men and from 5.4 ± 1.1 to 6.6 ± 0.5 km/h (*p* < 0.01) in women. The sex difference in running speed remained unchanged at 19.8% ± 4.8% (*p* > 0.05). In Spartathlon, the running speed was stable over time at 10.8 ± 0.7 km/h for men and 8.7 ± 0.5 km/h for women (*p* > 0.05). The sex difference remained unchanged at 19.6% ± 2.5% (*p* > 0.05).

**Conclusions:**

These results suggest that for both Badwater and Spartathlon, (a) female participation increased, (b) the fastest finishers were approximately 40 to 45 years, and (c) the sex difference was at approximately 20%. Women will not outrun men in both Badwater and Spartathlon races. Master ultramarathoners can achieve a high level of performance in ultramarathons greater than 200 km under extreme conditions.

## Background

In the past two decades, the field of ultra-endurance events defined as performance exceeding 6 h [1] has widened to a new arena in the sport of long-distance performances in particular ultra-running [2-4] and ultra-triathlon [5-7]. Today, more than a hundred thousand of ultramarathoners compete in more than a thousand races held annually around the world [8].

In recent years, the growth of ultra-endurance sports has drawn increased attention to investigate participation and performance trends [2,9,10]. A major focus of research in endurance sports was the sex difference in performance [11]. In the analysis of the world’s best times of female marathoners from 1980 to 1996, women were approximately 11% slower than men, although there was initially a considerable improvement in female running speed in the 1980s [12]. A similar sex difference for running speed has been found when running times between 100 m and 200 km were compared. Women were generally running approximately 12.4% slower than men [13]. In 160-km ultramarathons, the performance of the fastest women remained approximately 20% slower than that of the fastest men after an initial improvement relative to men throughout the 1980s [2]. In the shorter 100-km distance in the ‘100 km Lauf Biel’, the running times of the annual top ten finishers remained stable from 1998 to 2010 for women, while running times significantly increased for the annual top ten men [10]. The trend in sex difference was narrowing over time and averaged to 22% [10].

In both endurance and ultra-endurance events, a high-level performance seemed to be maintained until the age of 30 to 40 years, followed by a modest decrease until the age of 50 to 60 years, with a progressively accelerated decline after the age of 70 to 75 years [14-22]. The study of Hoffman [9] showed that peak running speed of the top performers in a 161-km ultramarathon was achieved by athletes in the age of upper 30 to 40 years. These results suggested an age shift into higher age in elite ultramarathon running in comparison with elite marathon running, where the fastest running speed were achieved by athletes of approximately 30 years of age [19].

Among the ultra-running events, the ‘Badwater’ [23] in the USA and the ‘Spartathlon’ [24] in Greece, Europe are recognized as two of the toughest running events worldwide. The common feature of both nonstop races is the distance of more than 200 km. The Badwater is characterized by its environmental conditions with extreme high temperatures and large altitude differences to overcome, while the Spartathlon is well known for its cut-off regulations with strict time limitations, apart from its unique historical background. To date, no study investigated the participation and running speed of female and male ultramarathoners competing in races longer than 200 km under extreme conditions such as heat and time limit. Therefore, the aims of the present study were to investigate (1) participation and performance trends and (2) the age of peak running speed in ultramarathons of more than 200-km race distance. Based upon present findings, we hypothesized (1) an increase in participation and an improvement in performance and (2) an age of peak ultramarathon performance between 30 and 40 years.

## Methods

The data set for this study was obtained from the race websites of Badwater [23] and Spartathlon [24]. Running speed and age of all female and male finishers in Badwater and Spartathlon between 2000 and 2012 were analyzed. The study was approved by the Institutional Review Board of St. Gallen, Switzerland, with waiver of the requirement for informed consent given that the study involved the analysis of publicly available data.

### Badwater

The event was established as an official foot race in 1987 with five successful US participants. Since 1989, the race starts at Badwater, the lowest elevation in the Western Hemisphere at 85 m below sea level and finishes at the Mt. Whitney Portals at nearly 2,530 m. Badwater covers a total distance of 217 km (135 miles) of highway nonstop across the Death Valley in California, USA. The course includes a total of 3,962 m of cumulative vertical ascent and 1,433 m of cumulative descent. During the race in Death Valley in mid-July, the average daily high temperature reaches 46.9°C, and temperatures over 50°C are common [25]. No aid stations are provided along the Badwater course, so runners rely on their own support crew to pass across the Death Valley. The number of participants is limited to 90 competitors.

### Spartathlon

The Spartathlon starting in 1982 is a nonstop foot race covering a total distance of 246 km (152 miles) from Athens to Sparta in Greece, Europe. Since 1983, the race has been continuously held each September. The course includes elevations which range from sea level to 1,200 m. The cumulative gain of elevations is approximately 1,650 m and the route runs on tarmac road, trail, or mountain footpath. The weather conditions during the race are typically changing between warm temperatures with about 27°C during the day and cold temperatures with about 5°C during the night. Aid stations are placed every 3 to 5 km, providing competitors with water and food. Each of the 75 race control points has its own time limitations and runners arriving later than the official closing time will be eliminated from the race. Nowadays, the number of applicants for the Spartathlon exceeds the limit of 350.

### Data analysis

Due to overall low participation and partially missing data in earlier years, only data between 2000 and 2012 were included for data analysis. In order to increase comparability of data, running times were converted to running speed prior to analysis. Converting was done using the equation running speed (km/h) = running distance (km) / running time (h). The changes in the performance and in the age of peak performance across years for both men and women were analyzed by examining the running speed and age of the annual top five female and male finishers from 2000 to 2012. The sex difference in performance between women and men was calculated using the equation sex difference (%) = [running speed in women (km/h) − running speed in men (km/h)] / running speed in men (km/h) × 100.

### Statistical analysis

In order to increase the reliability of the data analyses, each set of data was tested for normal distribution and for homogeneity of variances prior to statistical analyses. Normal distribution was tested using a D’Agostino and Pearson omnibus normality test, and homogeneity of variances was tested using a Levene’s test in case of two groups and with a Bartlett’s test in case of more than two groups. To find the significant changes in the development of a variable across the years, linear regression was used. To find significant differences between two groups, a Student’s *t* test was used in case of normal distributed data with additional Welch’s correction in case of significantly different variances between the analyzed groups and a Mann–Whitney test was used in case of not normal distributed data. To compare performance of men and women between the two races, a year-by-year analysis was performed using a two-way-analysis of variance with subsequent Bonferroni *post hoc* analysis. Also the interaction between the type of competition and time (years) on performance in genders was analyzed using a two-way-ANOVA (competition × time). Statistical analyses were performed using IBM SPSS Statistics (Version 19, IBM SPSS, Chicago, IL, USA) and GraphPad Prism (Version 5, GraphPad Software, La Jolla, CA, USA). Significance was accepted at *p* < 0.05 (two-sided for *t* tests). Data in the text are given as mean ± standard deviation or SD.

## Results

Between 2000 and 2012, data were available from 663 men and 183 women finishing Badwater and from 1,157 men and 141 women finishing Spartathlon.

### Participation trends

**Figure 1:**
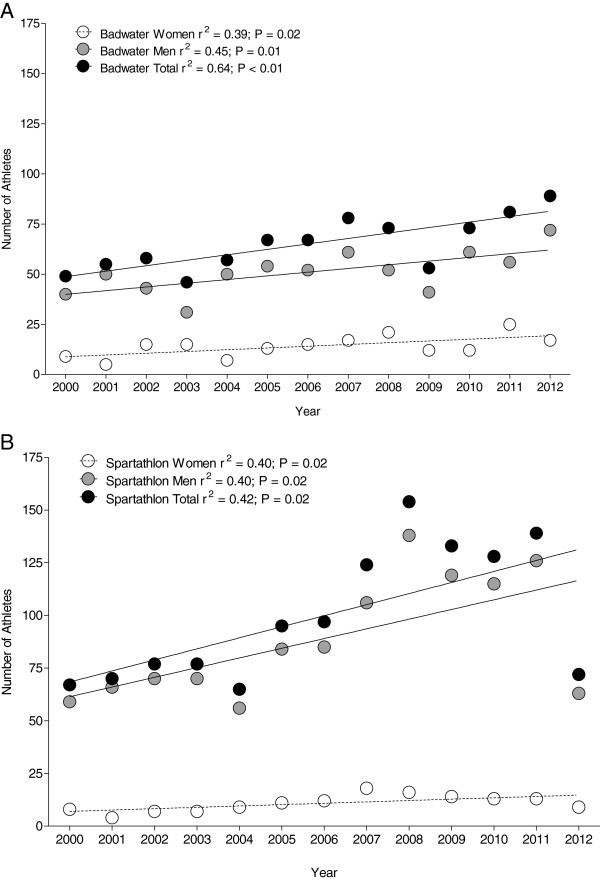
Annual number of female, male and overall finishes in Badwater (A) and Spartathlon (B).

During the 13-year period, the annual overall percent finishes averaged 79% (ranging from 62% to 93%) for Badwater and 43% (ranging from 39% to 50%) for Spartathlon. Between 2000 and 2012, the number of both female and male finishes increased in both Badwater (Figure 1A) and Spartathlon (Figure 1B). A mean of 51 ± 11 men finished annually in Badwater and 89 ± 23 men in Spartathlon. In women, the mean number of annual finishers was on average 14 ± 5 athletes in Badwater and 11 ± 4 in Spartathlon. There was a significant increase in female participation in Badwater from 18.4% to 19.1% (maximum of 30.9% in 2011) and in Spartathlon from 11.9% to 12.5% (maximum of 14.5% in 2007). Women accounted on average for 21.5% ± 6.9% in Badwater and 10.8% ± 2.3% in Spartathlon of the overall finishes.

### Age of finishers in Badwater and Spartathlon

In men, the mean age of all finishers was higher in Badwater (46.5 ± 9.3 years) compared to Spartathlon (44.8 ± 8.2 years) (*p* < 0.01). The mean age of female finishers in both races was similar with 43.0 ± 7.5 years in Badwater and 44.5 ± 7.8 years in Spartathlon (*p* > 0.05).

**Figure 2:**
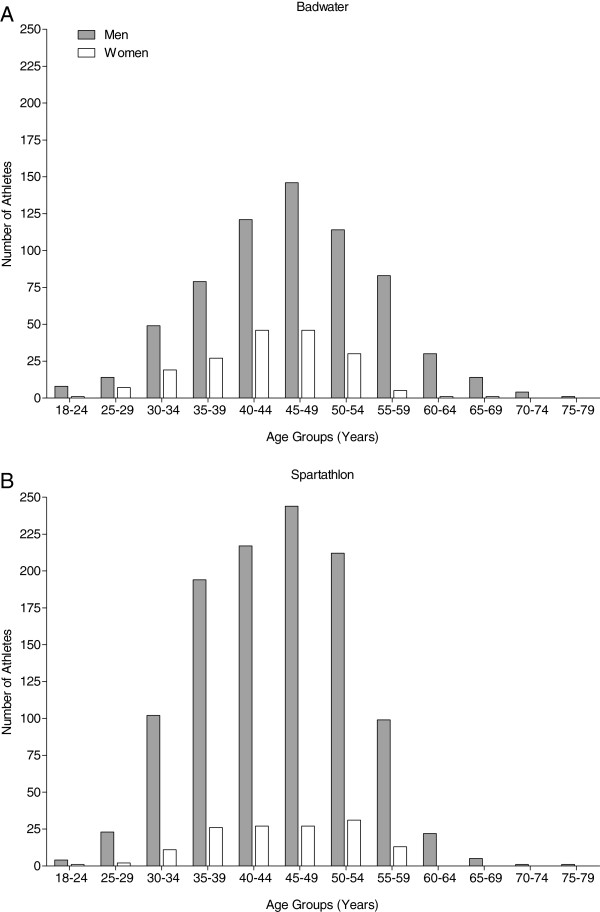
Male and female finishes in Badwater (A) and Spartathlon (B) per age group.

The age distribution of finishers is presented in Figure 2 for Badwater (Figure 2A) and Spartathlon (Figure 2B). In men, the largest participation was in athletes in the age group of 45 to 49 years in both races. For women, the largest participation was in age groups of 40 to 44 and 45 to 49 years in Badwater; while in Spartathlon, the largest participation was in the age group of 50 to 54 years. Master athletes (>40 years of age) represented 76% (642/846) of the total field in Badwater and 71% (899/1,262) in Spartathlon.

**Figure 3:**
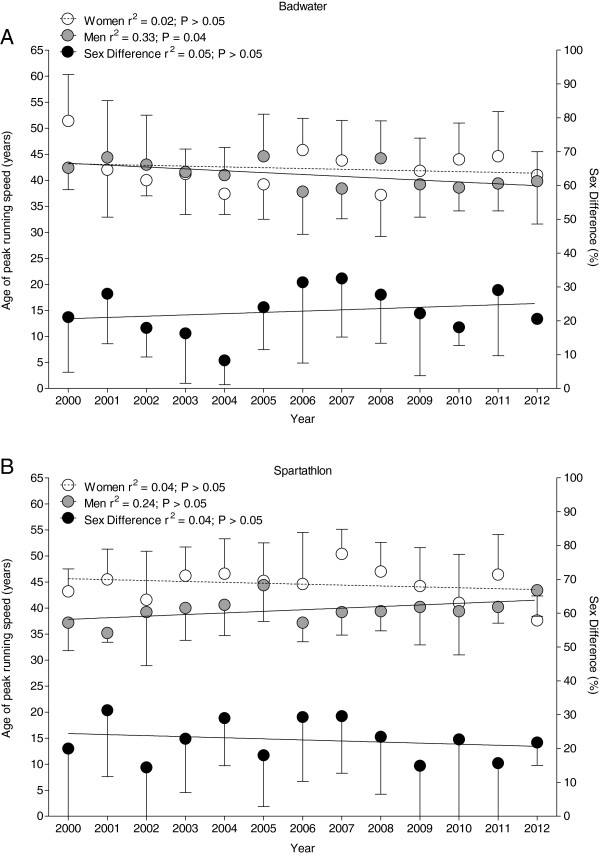
Age of the annual top five women and men in Badwater (A) and Spartathlon (B).

Over the years, the age of the annual five fastest men decreased in Badwater from 42.4 ± 4.2 years in 2002 to 39.8 ± 5.7 years in 2012 (Figure 3A). For women, the mean age of the annual five fastest finishers remained unchanged at 42.3 ± 3.8 years in Badwater. In Spartathlon (Figure 3B), the age of the annual five fastest finishers was unchanged at 39.7 ± 2.4 years for men and 44.6 ± 3.2 years for women.

### Running speed of the annual top five finishers

**Figure 4:**
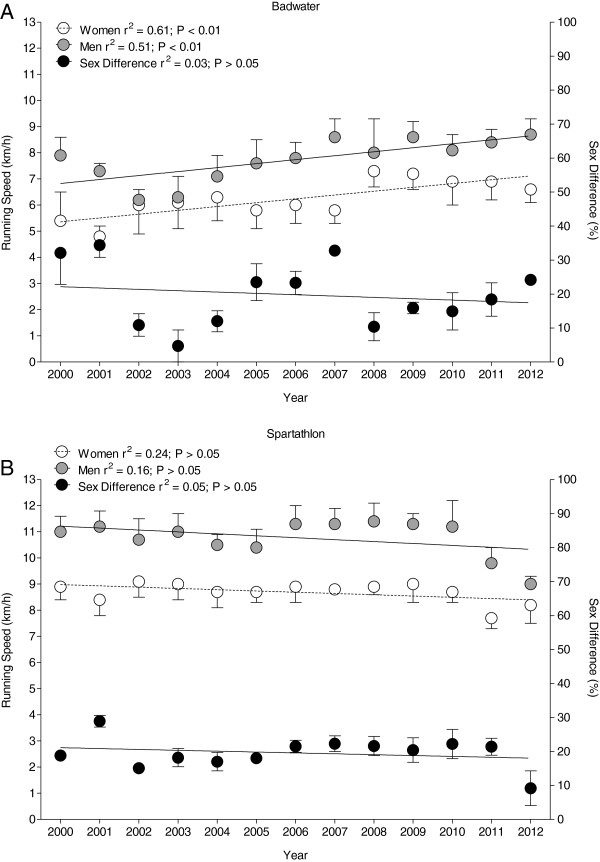
Running speeds of the annual top five women and men in Badwater (A) and Spartathlon (B).

Regarding the annual top five finishers, the mean running speed was lower in Badwater compared to Spartathlon (*p* < 0.001). In Badwater, both women and men became faster over years (Figure 4A). Running speed in men increased from 7.9 ± 0.7 km/h in 2000 to 8.7 ± 0.6 km/h in 2012. In women, running speed increased from 5.4 ± 1.1 km/h to 6.6 ± 0.5 km/h. The sex difference in running speed remained stable at 19.8% ± 4.8%. In Spartathlon running speed remained unchanged at 10.8 ± 0.7 km/h for men and 8.7 ± 0.5 km/h for women (Figure 4B). The sex difference in running speed remained unaltered at 19.6% ± 2.5%.

**Figure 5:**
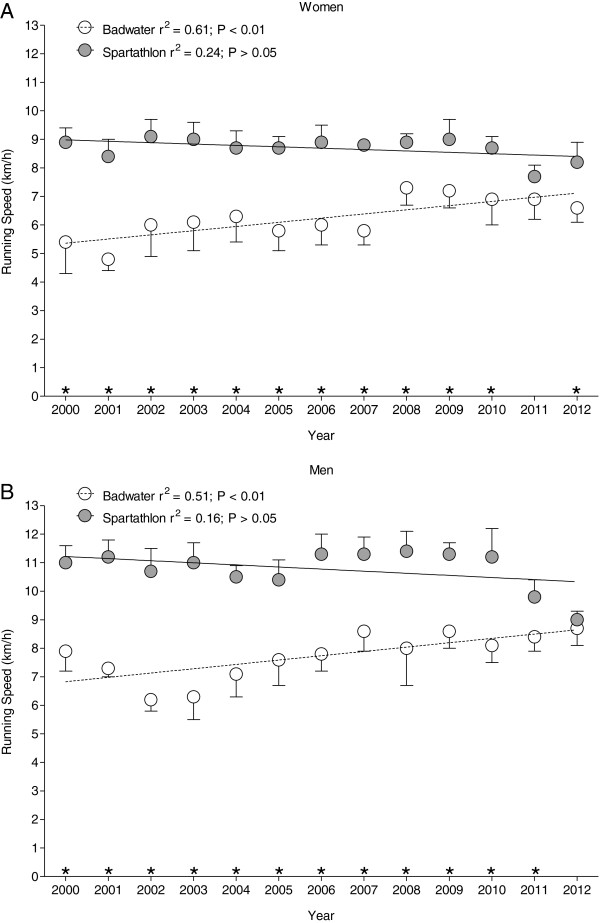
**Comparison of running speed between Badwater and Spartathlon for annual top five.** Comparison of running speed between Badwater and Spartathlon for the annual top five women (**A**) and men (**B**). An *asterisk* indicates years in which the running speed in Badwater was significantly different (*p* < 0.01) from the running speed in Spartathlon.

The running speed of the annual top five finishers was significantly (*p* < 0.01) faster in Spartathlon compared to Badwater for women and men, with the exception of 2011 in women (Figure 5A) and 2012 in men (Figure 5B). The analysis of interaction between type of competition and time (years) on running speed showed for women a highly significant (*F* = 4.1; *p* < 0.01) interaction between type of competition and time accounting for 7.8% of the total variance, whereas the type of competition accounted for 69.2% (*F* = 436; *p* < 0.01) and time accounted for 6.7% (*F* = 3.5; *p* < 0.01) of the total variance. In men, the interaction between the type of competition and time accounted for 9.9% of the total variance (*F* = 7.4; *p* < 0.01), whereas type of competition accounted for 71.6% (*F* = 642.1; *p* < 0.01) and time accounted for 7.0% (*F* = 5.2; *p* < 0.01) of the total variance.

## Discussion

This study intended to investigate participation and performance trends and to determine the age of peak running speed in ultramarathons of more than 200-km race distance. Based upon existing literature, an increase in participation, an improvement in performance, and an age of peak ultramarathon performance between 30 and 40 years were hypothesized. The main findings for both races were that (1) the female participation increased over time, (2) the fastest finishers were approximately 40 to 45 years of age, and (3) the sex difference was at approximately 20% unchanged over years.

### Participation trends

Concerning the total numbers of finishes over the history, it appears reasonable that in Badwater considerably fewer athletes competed since the qualifying standards were higher [23] than in Spartathlon [24]. Another limiting factor may be attributed to the greater financial expenses to participate in the Badwater because there are high additional costs for logistics and the necessity of a supporting team [23]. Across the history of Badwater race, the annual percentage of finishes averaged to 79% versus 43% in the Spartathlon. This relative low percentage of finishes in the Spartathlon may reflect the extraordinary demands on running speed due to rigorous cut-off limits in the Spartathlon.

Regarding the sex-specific participation, female ultramarathoners accounted on average for approximately 22% in Badwater versus approximately 11% in Spartathlon. Since women were generally slower in ultramarathon running than men [2-4], the cut-off limits in the Spartathlon could have a greater influence on women for a successful finish. Present findings were in accordance with other studies regarding the fact of a relatively low female participation in ultramarathon in comparison to shorter running events such as a marathon [3,4,9,10]. For instance, in the 100 km Lauf Biel in Switzerland, female participation was at approximately 13%. Hoffman reported a female participation of approximately 20 to 22% in 161-km ultramarathon events held in North America, which comes close to the female participation in Badwater. Men are over-represented in sports [26]. Generally, men were running faster than women [27], and the sex difference in relative performance can partially be attributed to men’s greater training motivation [26]. The motivation to train and compete seems different between women and men. The most popular modern male sports require the skills needed for success in male-male physical competition [28]. In contrast, female ultramarathoners were described as task-oriented, internally motivated, healthy, and financially conscious individuals [29].

The three five-year age groups between 40 and 54 years accounted for the largest participation regardless of sex and race location. This age-related finding, together with the fact of numerous finishes from athletes aged 40 years and older in Badwater (76%) and in Spartathlon (71%), is comparable with the results of other studies investigating the age distribution in ultra-running events [4,9,10]. Our data may reflect a lifestyle trend in sports activities towards ultramarathon. Younger athletes are increasingly more attracted by technical sports with higher intensity [10,30,31]. The higher participation of elderly athletes may be due to social and psychological factors such as spare time resources connected with an increase in the competitive spirit [32]. Hoffman and Fogard [4] described 161-km ultramarathoners as master runners at a mean age of 44.5 ± 9.8 years with a range between 20 and 72 years. These runners were generally men (80.2%), married (70.1%), and had higher education with bachelor’s (43.6%) or graduate (37.2%) degrees. A further reason for the high percentage of older finishers across the studied history of both races could be related to the logistic demands, particularly of Badwater. The extraordinary distance over 200 km of both events may require an enormous volume of training over years. In ultramarathoners, previous experience is of importance for a successful race outcome [5].

### Performance trends

The present study adds valuable results considering the change in demographic aspects of participation and performance in two extraordinary strenuous ultramarathons of more than 200 km in length. The improvement in running speed in Badwater might be attributed to a progress in the supporting teams and to an accumulation of pre-race experience [33,34]. The increase in the number of competitors and finishers might reflect that faster and more experienced runners enter this race [35-37].

In Spartathlon, however, athletes were not able to improve running speed. The stable running speeds in Spartathlon over the same time span could be attributed to the character of this race which is more a pure running event and has not to rely so much on external support in comparison to the Badwater [23]. Analyses of Hoffman and Wegelin [9] and Knechtle et al. [10] could not detect a clear improving or declining performance trend for the top ultramarathoners in terms of running speeds. Concerning Badwater, our data give a different impression due to a significant improvement of race speed regardless of sex. On the whole, independent of sex, considerably faster running speeds were achieved by the annual top five finishers in Spartathlon in comparison to the annual top five runners in Badwater. Different speed levels reflect the different physical demands of either race. Interestingly, in 2011 the running speeds in women narrowed. One possible explanation for this finding could be a lower top five elite field in the Spartathlon in that year. Further adverse race conditions may slow down women running speed in the Spartathlon in 2011.

### Sex difference in ultra-running performance

In both Badwater and Spartathlon, the sex difference in running performance was at approximately 20%. Generally, the sex difference in endurance performance is at approximately 11 to 12% [12,13]. The analysis of world best times in 1,500 m and marathon running from 1980 to 1996 revealed that men were approximately 11% faster than women [12]. In a further widespread investigation of running distances between 100 m and 200 km, men were approximately 12% faster compared to women accompanied by the tendency that longer distances were associated with greater sex differences in endurance performance [13].

Regarding the sex difference in elite ultramarathon running, our data are in accordance with the previous studies. Hoffman [2] reported for 161-km ultramarathoners that the fastest women were approximately 20% slower than the fastest men. In a comparable race, the 100 km Lauf Biel in Switzerland, the top ten women were approximately 22% slower than the top ten men [10]. In the current discussion about sex difference in ultramarathon running, exceptional findings do exist supporting the assumption that women could outperform men in ultra-endurance sports. For instances, Bam et al. [38] compared long-distance performances in both men and women and observed that men were faster over distances up to marathon, but this was not evident in a 90-km ultramarathon. Also Knechtle et al. [39] described a female overall winner in a multi-stage ultramarathon.

Looking upon the sex-related running speed difference between the top elite runners over the 2000 to 2012 period, it is notable that in the 2002 and 2003 Badwater races, the sex difference in top five running speed was close to zero. Moreover, female winners even outperformed male winners in these years. In 2003, the hottest climate conditions have ever been registered in Death Valley with the highest drop off rate of 37% was published for this year [23]. Thus, these results from 2002 to 2003 appeared to reflect a possible better coping against heat among women. Jacob et al. [40] reported that increasing ambient temperatures had less adverse effects on running speed for women than for men in 161-km ultramarathons. Our findings do underline the assumption that top running speed of women does not converge with or even surpass those of men apart from exceptional women top running speed as seen in the Badwater in 2002 and 2003.

The higher sex difference in performance in these ultramarathoners might be explained by differences in anthropometry, physiology, training, and motivation between women and men. Ultramarathon running leads to a decrease in skeletal muscle mass [41]. Since male ultramarathoners [42] have a higher skeletal muscle mass compared to female ultramarathoners [43], the lower muscle mass in women might be a limiting factor for ultramarathon performance. However, physiology still plays an inevitable roll that sex differences probably will continue in the future. Sex difference in performance is considered due primarily to inherent gender-specific differences in body composition and oxygen transport capacity [12,44-46]. Considering training and motivation, female and male ultra-endurance athletes were trained for about the same weekly hours, but men were training faster than women [47,48]. Furthermore, men pay closer attention to male sports than women do and male champion athletes obtain a higher status [28].

### The age of peak ultra-running performance

From 2000 to 2012, for both men and women, the age of the annual top five finishers remained at 39 to 45 years in Badwater and Spartathlon. Women achieved their peak running speed at higher age than men in either event. Our data were comparable with other studies in ultramarathon running events. By investigating the top ten runners in the 100 km Lauf Biel, the age of the best performance was found at approximately 39 to 40 years [10]. Across the history of the 161-km ‘Western State Endurance Run’ in North America, the average age of the top five finishers gradually increased from the early 30s to the upper 30s [9].

Furthermore, existing literature and our findings suggest that with the increasing length of running events, athletes achieved their peak of running speed at higher age than in shorter running events [9,49,50]. The analysis of the ‘New York City Marathon’ [51] documented that among the top fifty performers, no runner was older than 39 years independent of gender. The present data of Badwater and Spartathlon are also consistent with the findings of Schulz and Curnow [50], reporting a relationship between the age of peak performance and specific events already in the 1980s. Taken as a whole, the present and previous findings [9,10] evidenced that among the top ultramarathon, elite runners were master athletes defined as participants older than 35 years of age [52]. Usually, however, these older athletes are considered to have either finished their formal competitive careers, to be ‘weekend warriors’ who sporadically train and compete, or to have start again dynamic exercises after a long time span of physical inactivity [53]. Thus, present and other findings appear to suggest that the definition of master runners need to be revised for ultramarathoners.

### Limitations

This cross-sectional data analysis is limited since aspects such as anthropometry [35-37], the physiology [54,55], and the training [35-37] of the runners, their previous experience [34-37], their pacing strategy [56], the environmental conditions [57], and both nutrition [58-60] and fluid intake [61,62] were not considered.

## Conclusions

To summarize, female participation increased in ultramarathons of more than 200 km such as Badwater and Spartathlon; the fastest finishers were approximately 40 to 45 years of age, and the sex difference was at approximately 20%. These results suggest that women will not outrun men in the near future in ultramarathons of greater than 200 km in length such as Badwater and Spartathlon. Master ultramarathoners can achieve high level of performance in ultramarathons greater than 200 km under extreme conditions such as heat and time limit. Apart from the influence of other limiting factors on the running speed in ultramarathon such as physiological and anthropometric characteristics, further studies need to investigate what motivates female and male master ultramarathoners to compete in these extreme races. The definition of master runners as athletes aged 35 years and older needs to be revised for ultramarathoners competing in races of greater than 200 km. Future studies need to investigate the age of peak ultramarathon performance in longer running distances.

## Availability of supporting data

The data sets supporting the results of this article are available in http://spartathlon.gr/ and http://badwater.com/.

## Competing interests

The authors declare that they have no competing interests.

## Authors’ contributions

KFE drafted the manuscript. BK participated in the conception and design of the study, helped to draft the manuscript, and participated in the analysis and interpretation of data. CAR performed the analysis of data and participated in the interpretation of data. PK helped in the acquisition of data. RL and TR performed the interpretation of data and manuscript preparation. All authors read and approved the final manuscript.
